# Cytological and Proteomic Analysis of Wheat Pollen Abortion Induced by Chemical Hybridization Agent

**DOI:** 10.3390/ijms20071615

**Published:** 2019-04-01

**Authors:** Shuping Wang, Yingxin Zhang, Zhengwu Fang, Yamin Zhang, Qilu Song, Zehao Hou, Kunkun Sun, Yulong Song, Ying Li, Dongfang Ma, Yike Liu, Zhanwang Zhu, Na Niu, Junwei Wang, Shoucai Ma, Gaisheng Zhang

**Affiliations:** 1 Hubei Collaborative Innovation Center for Grain Industry/Hubei Key Laboratory of Waterlogging Disaster and Agricultural Use of Wetland/College of Agriculture, Yangtze University, Jingzhou 434000, China; zhangyingxin1985@126.com (Y.Z.); fangzhengwu88@163.com (Z.F.); houzehao1994@126.com (Z.H.); 201772390@yangtzeu.edu.cn (K.S.); madf@yangtzeu.edu.cn (D.M.); 2 College of Agriculture, Northwest A&F University, Yangling 712100, China; ymzhang2017@163.com (Y.Z.); songqilu1234@163.com (Q.S.); sylbl1986@163.com (Y.S.); qiuxuewuying@163.com (Y.L.); niuna@nwsuaf.edu.cn (N.N.); wjw@nwsuaf.edu.cn (J.W.); mashoucai@nwsuaf.edu.cn (S.M.); 3 Institute of Genetics and Developmental Biology, Chinese Academy of Sciences, Beijing 100101, China; 4 Food Crops Institute, Hubei Academy of Agricultural Sciences, Wuhan 450064, China; liuyike@webmail.hzau.edu.cn (Y.L.); zhuzhanwang@163.com (Z.Z.)

**Keywords:** CHA-SQ-1, cytomorphology, pollen abortion, proteomics, wheat

## Abstract

In plants, pollen grain transfers the haploid male genetic material from anther to stigma, both between flowers (cross-pollination) and within the same flower (self-pollination). In order to better understand chemical hybridizing agent (CHA) SQ-1-induced pollen abortion in wheat, comparative cytological and proteomic analyses were conducted. Results indicated that pollen grains underwent serious structural injury, including cell division abnormality, nutritional deficiencies, pollen wall defect and pollen grain malformations in the CHA-SQ-1-treated plants, resulting in pollen abortion and male sterility. A total of 61 proteins showed statistically significant differences in abundance, among which 18 proteins were highly abundant and 43 proteins were less abundant in CHA-SQ-1 treated plants. 60 proteins were successfully identified using MALDI-TOF/TOF mass spectrometry. These proteins were found to be involved in pollen maturation and showed a change in the abundance of a battery of proteins involved in multiple biological processes, including pollen development, carbohydrate and energy metabolism, stress response, protein metabolism. Interactions between these proteins were predicted using bioinformatics analysis. Gene ontology and pathway analyses revealed that the majority of the identified proteins were involved in carbohydrate and energy metabolism. Accordingly, a protein-protein interaction network involving in pollen abortion was proposed. These results provide information for the molecular events underlying CHA-SQ-1-induced pollen abortion and may serve as an additional guide for practical hybrid breeding.

## 1. Introduction

In wheat plants, pollen develops in the anther, a highly specialized organ. Sporogenous cells (center of anther locule) give rise to microsporocyte. The microsporocyte undergoes two meiotic divisions, developing into a tetrad of haploid microspores (tetrad stage). Then the microspores are released, and each consists of a central nucleus (early-uninucleate stage). These microspores grow and undergo cell polarization until the nucleus is adjacent to the wall, and a single vacuole dominates the intracellular space (later-uninucleate stage). The polarized cell then divides to form one large vegetative cell and one small generative cell (binucleate stage). Later, this bicellular system produces tricellular pollen (a vegetative cell and two sperm cells; trinucleate stage), forming mature pollen grain [1,2,3,4]. Therefore, the development of mature pollen grain follows a tightly controlled sequence of events within the anther. Once this sequence is broken, pollen abortion occurs. In previous study, SQ-1 is an effective CHA for wheat and can impair the production and release of viable pollen [2]. Previous research studies on CHA-SQ-1-induced male sterility were mainly concentrated on reactive oxygen metabolism, aliphatic metabolism, and DNA methylation [5,6,7], the genetic and molecular mechanisms of CHA induced wheat pollen sterility still needs to be further elucidated.

In plant, pollen grains are highly useful model for investigating the molecular mechanisms underlying cell differentiation, polar cell growth, cell-to-cell communication, and cell fate determination. Additionally, the development of mature pollen grain was followed by a tightly controlled sequential process within anthers. Once this process is disturbed, pollen abortion occurs [2]. In the past two decades, pollen function specialization has been studied using biochemistry, functional genomics, and molecular genetics. More than 150 genes have been found to regulate pollen development, as indicated by reverse genetics, and some of these genes have been functionally characterized and expressed at specific stages of pollen maturation [8]. Additionally, a large number of transcripts expressed during pollen development have been identified during pollen development, which encode proteins involved in heat shock, cytoskeleton, pollen cell wall, allergen, cell division, signal transduction, pectin, carbohydrate, and energy metabolism [9]. Understanding the exact mechanisms underlying pollen development and fertilization may facilitate more advanced crop breeding and engineering. These issues have been investigated in higher plants in studies of male-sterile mutants [10,11,12], factors affecting pollen germination and tube growth [13,14], and the molecular mechanisms underlying self-incompatibility [15]. More importantly, pollen development is known to be very sensitive to abiotic stress [1]. In particular, high temperature [16,17], low temperature [18,19], drought [20], and water-stress [21] can result in male sterility in crops, including *Hordeum vulgare* [22], *Oryza sativa* [23], *Triticum aestivum* [18]. Meanwhile, proteomics is a powerful approach to study the molecular processes of pollen development, and also a powerful complement to the whole genome sequencing [24]. Based on the proteins identified in this work, a detailed pathway was successfully constructed and protein-protein interactions were more clearly understood [25]. To date, there has been an increased application of proteomics approaches to study the pollen reproduction or pollen responses to abiotic stress, for example, *Lycopersicon esculentum* (Response to heat-stress and ethylene) [18], *Oryza sativa* (Response to high temperature-stress) [23], *Triticum aestivum* (Response to drought-stress) [26]. Thus, proteomics has been extensively used to investigate the protein expression pattern in pollen development under several abiotic stresses [16,27].

Recently, there have been some progress in studies of CHA-SQ-1-induced male sterility of wheat, for example, metabolism [5,6,28], DNA methylation [7], cell morphology [2,3], transcriptome [29], and proteomic [30,31] of flag leaf [30], floret [32], and anther [2,4,31]. These results provide necessary theoretical basis for studying the mechanism of CHA-SQ-1 induced male sterility. The objective of this study was to uncover the cytological and biochemical mechanisms of CHA-SQ-1 induced pollen abortion in wheat. Towards this objective, a comprehensive analysis of pollen grain cytomorphology and proteome was performed. CHA-SQ-1 impaired pollen maturation and resulted in complete pollen sterile. As expected, 60 identified differential abundant proteins (DAPs) in this study did play important roles in cell growth and division, stress response, carbohydrate and energy metabolism, and protein metabolism.

## 2. Results

### 2.1. Cytological Changes in Pollen Abortion Induced by CHA-SQ-1 in Wheat

**Figure 1:**
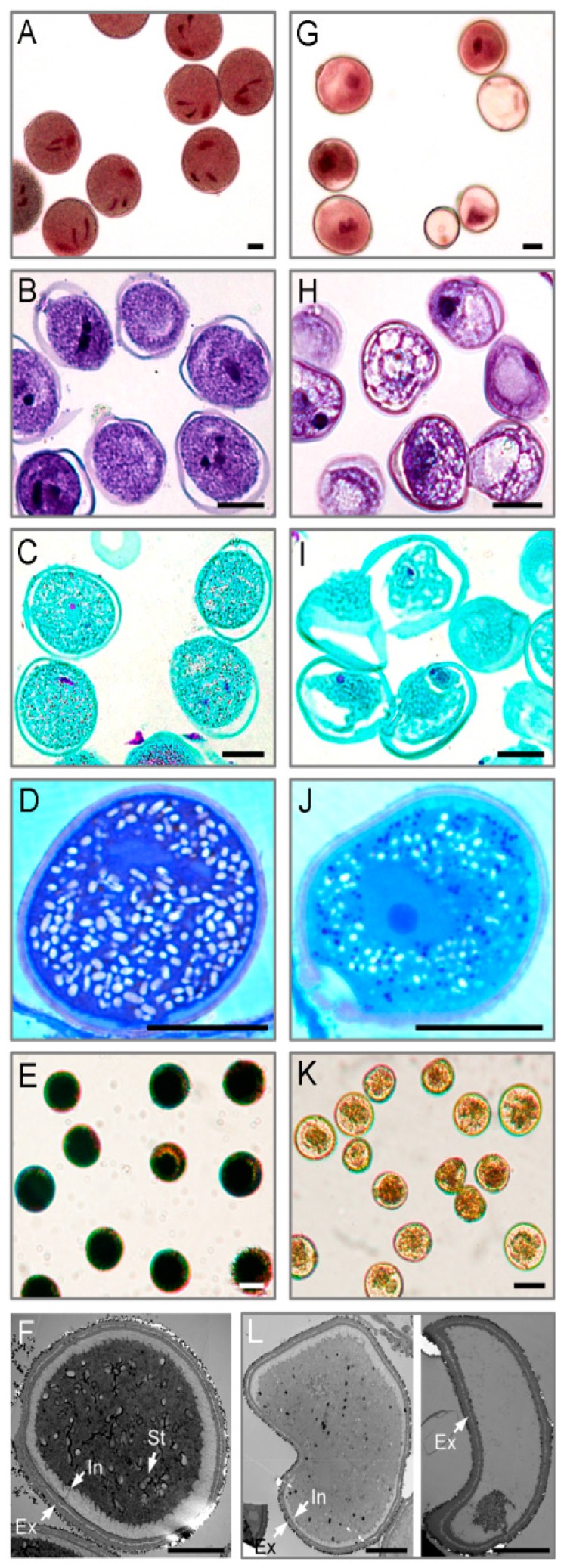
Comparison of pollen grain from the control (**A**–**F**) and CHA-SQ-1-treated wheat plants (**G**–**L**) during pollen maturation. (**A**,**G**) 1% acetocarmine staining. (**B**,**H**) Ehrlich’s hematoxylin staining. (**C**,**I**) safranin O/fast green staining. (**D**,**J**) CBB-R250 staining. (**E**,**K**) I_2_-KI staining. (**F**,**L**) Transmission electron micrograph. Ap, germination aperture; Ex, exine; In, intine; St, starch granule; Bars: 20 μm (**A**–**E**,**G**–**K**), 10 μm (**F**,**L**).

Morphological differences of pollen grains between control and CHA-SQ-1-treated plants were revealed by microscopic observation (Figure 1). The pollen grains of controls showed two sperm nuclei, detectable nuclear nutrients (Figure 1A), and a full complement of storage materials (Figure 1B and C) fostering pollen viability and facilitating function, which include proteins (Figure 1D) and starch granules (Figure 1E). However, the pollen grains of CHA-SQ-1 treated plants showed abnormal development (Figure 1G) and accumulated less nutritional material in the cytoplasm (Figure 1H–K). Unlike control pollen grains, which showed strong staining, none of the treated pollen grains were deeply stained with iodine-potassium iodide (2% I_2_-KI; Figure 1E,K), which indicated that the plants were 100% pollen sterile.

Detailed examination of treated and control pollen grains was performed to identify defects in cytological structural using transmission electron microscopy (TEM, Figure 1F,L). TEM analysis showed that the control pollen grains had fairly dense cytoplasm and normal pollen walls with distinguishable exine and intine layers (Figure 1F). Although the walls of pollen grains from CHA-SQ-1-treated plants seemed to have normal exine, the pollen grains had less dense cytoplasm and often empty chambers, and the intine was thin or undetectable (Figure 1L).

These results indicated that pollen grains were impaired by CHA-SQ-1, resulting in abnormal pollen development and shape, reduced storage materials, defective pollen intine, and collapsed pollen grains.

### 2.2. Pollen Grain Proteomic Analysis of CHA-SQ-1-Treated Wheat Plants

**Figure 2:**
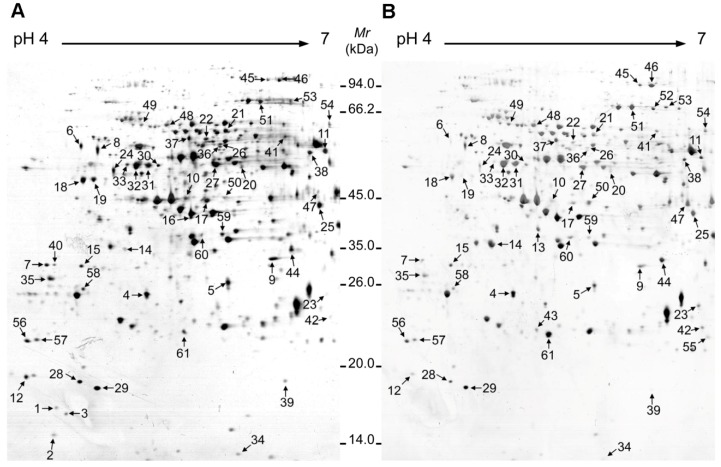
2-DE electrophoresis gels of pollen grain proteomes in the control (**A**) and CHA-SQ-1-treated wheat plants (**B**). Proteins were stained with CBB-G250. About 550 μg of protein was loaded onto IPG strips at pH 4–7 (17 cm, linear). SDS-PAGE was performed using 12% gels. Identified proteins are numbered on the gels.

To determine which pollen grain proteins changed in abundance in response to CHA-SQ-1-treatment, a proteomic study was performed using two-dimensional gel electrophoresis (2-DE) and MALDI-TOF/TOF MS. Three independent biological replicates were performed in this 2-DE experiment. Figure 2 shows a representative gel image of proteins extracted from control and CHA-SQ-1 treated plants, respectively. From a spot-to-spot comparisons and statistical analysis, a total of 61 protein spots exhibited at least 1.5-fold (*P* ≤ 0.01) difference in abundance between the control and CHA-SQ-1 treated plants (Figure 2 and Table S1). Of these, 19 spots were high-abundant and 42 spots were low-abundant in pollen grains from CHA-SQ-1-treated plants (Table S1).

### 2.3. Identification and Classification of DAPs

All 61 DAPs were further analyzed using MALDI-TOF/TOF MS, and 60 of them were successfully identified (Table S2). Of these, 57 were functionally annotated in the current database, but the remaining three identities were unnamed proteins (spot 58, spot 59, and spot 60; Tables S2 and S3). To annotate these proteins, their sequences were used as queries and NCBI was searched for homologues using BLASTP. The result was listed in Table S4. Homologues were defined as proteins sharing at least 80% positive identity with the target at the amino acid level, which was here considered indicative of similar function. In this way, protein homologues were divided into appropriate categories. However, about one third of the identified proteins were detected in multiple spots with different pIs or molecular masses (Table S2). This suggested the existence of isoforms and posttranslational modification. Similar results were also found in others [24,33]. Taken together, the 60 identities represented 49 unique proteins (Tables S2 and S4).

**Figure 3:**
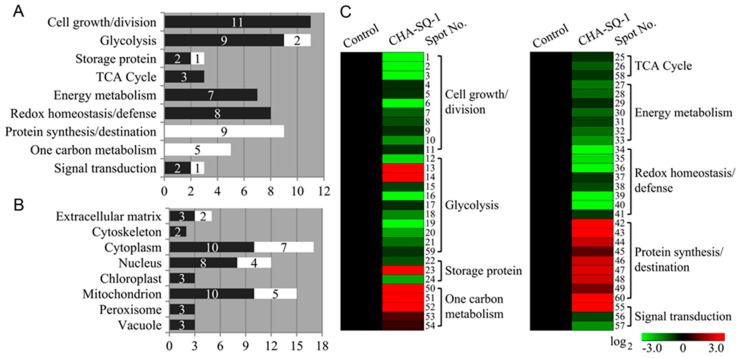
Functional classification, subcellular localization, and hierarchical clustering of the 60 DAPs in pollen grain of the control and CHA-SQ-1-treated wheat plants. The DAPs were divided into nine functional groups (**A**) and classified by predicted subcellular localization (**B**) using Euk-mPLoc 2.0 database (http://www.csbio.sjtu.edu.cn/bioinf/euk-multi-2/), BaCelLo database (http://gpcr.biocomp.unibo.it/bacello/pred.htm) and ESLpred database (http://www.imtech.res.in/raghava/eslpred2/). The black bars indicate high-abundant proteins and the white bars low-abundant proteins. (**C**) The hierarchical cluster analysis was conducted using the Genesis 1.7.6 procedure (Graz University of Technology, Austria, http://genome.tugraz.at/genesisclient/genesisclient_download.shtml) and the log_2_-transformed values of -fold change ratios listed in Table S1.

Furthermore, based on the metabolic and functional features of wheat pollen, all of these identities were classified into nine functional groups (Figure 3A,C; Table S2), including cell growth and division (18%, eleven low-abundant proteins in CHA-SQ-1 treatment), glycolysis (18%, nine low-abundant and two high-abundant proteins in treatment), protein synthesis and destination (15%, nine high-abundant proteins in treatment), redox homeostasis and defense (13%, eight low-abundant proteins in treatment), energy metabolism (12%, seven low-abundant proteins in treatment), one carbon metabolism (9%, five high-abundant proteins in treatment), storage protein (5%, two low-abundant and one high-abundant proteins in treatment), TCA cycle (5%, three low-abundant proteins in treatment), and signal transduction (5%, two low-abundant and one high-abundant proteins in treatment). An impressive 85% of these identified proteins were implicated in the first six functional groups, whereas the largest functional group consisted of proteins involving cell growth and division (18%) and glycolysis (18%), which were greatly affected by CHA-SQ-1 treatment. Further analysis of the abundance changes of each group revealed that proteins involved in protein synthesis and destination (15%), redox homeostasis and defense (13%), energy metabolism (12%), and one carbon metabolism (9%) were overrepresented, either in number or in expression level, suggesting that these processes were susceptible to CHA-SQ-1 treatment during pollen maturation.

The subcellular location of a protein can indicate its physiological function. Here, prediction showed that these differentially accumulated proteins were localized at the extracellular matrix (9%, three low-abundant and two high-abundant proteins in treatment), cytoskeleton (3%, two low-abundant proteins in treatment), cytoplasm (28%, ten low-abundant and seven high-abundant proteins in treatment), nucleus (20%, eight low-abundant and four high-abundant proteins in treatment), chloroplast (5%, three low-abundant proteins in treatment), mitochondrion (25%, ten low-abundant and five high-abundant proteins in treatment), peroxisome (5%, three low-abundant proteins in treatment), and vacuole (5%, three low-abundant proteins in treatment, Figure 3B). In order to visually portray the patterns of protein expression in all nine functional categories, hierarchical clustering of proteins was analyzed and a graphic was produced (Figure 3C).

### 2.4. Protein-Protein Interactions Network in CHA-SQ-1-Induced Pollen Abortion

**Figure 4:**
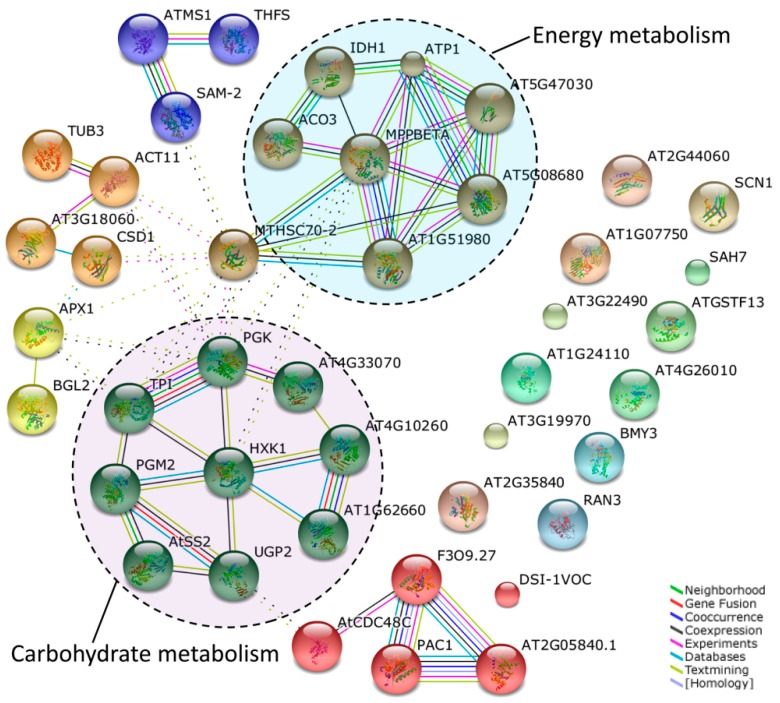
Analysis of protein interaction networks using the STRING system. TAIR homologous proteins among those identified here were mapped using STRING 10 software at a confidence of 0.4. All the homologous proteins are listed in Table S5. Abbreviations of the specific protein names in STRING network were presented in Table S6. Colored lines between the proteins indicate the various types of interaction evidence. The two clusters of protein nodes that interacted closely and frequently are indicated by circles. They include proteins involved in carbohydrate and energy metabolism.

**Figure 5:**
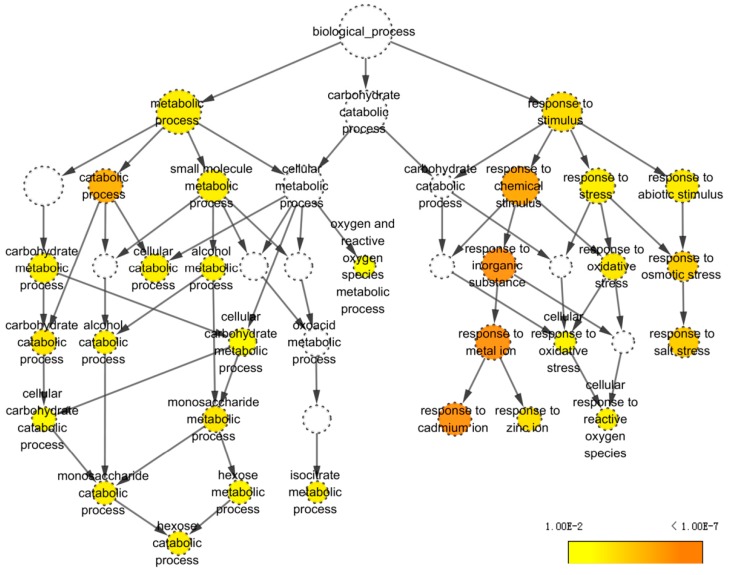
Biological pathway networks generated using the BiNGO plugin from Cytoscape tool. Homologous proteins were used here for GO analysis. Node size is shown as proportional to the number of proteins placed in the GO category. Color denotes the *P*-value of each enriched GO term (color scale, right bottom). White nodes are not enriched.

In living cells, proteins do not act as single entities. Rather, they form a network of functional interconnections that underlie the cellular processes. To determine how CHA-SQ-1 interacts with the wheat pollen grains and affect cell functions, identified proteins were annotated using Arabidopsis thaliana TAIR10 protein database (Table S5), the corresponding AGI codes were then used to generate an interactome on STRING (Figure 4) and BiNGO (Figure 5).

The STRING analysis revealed a protein association network (Figure 4). Specific protein names and abbreviations are given in Table S6. The two major clusters identified included proteins involved in carbohydrate and energy metabolism. CHA-SQ-1 treatment was found to inhibit the accumulation of a protein essential to carbohydrate metabolism, hexokinase (HXK1, gi|475536774, spot15). Heat shock 70 kDa protein (MTHSC70-2; gi|473970552, spot 48, and gi|379645201, spot 49) is the core protein of this network, and it interacts with many other clusters (carbohydrate metabolism, energy metabolism, cell growth/division, one carbon metabolism).

**Figure 6:**
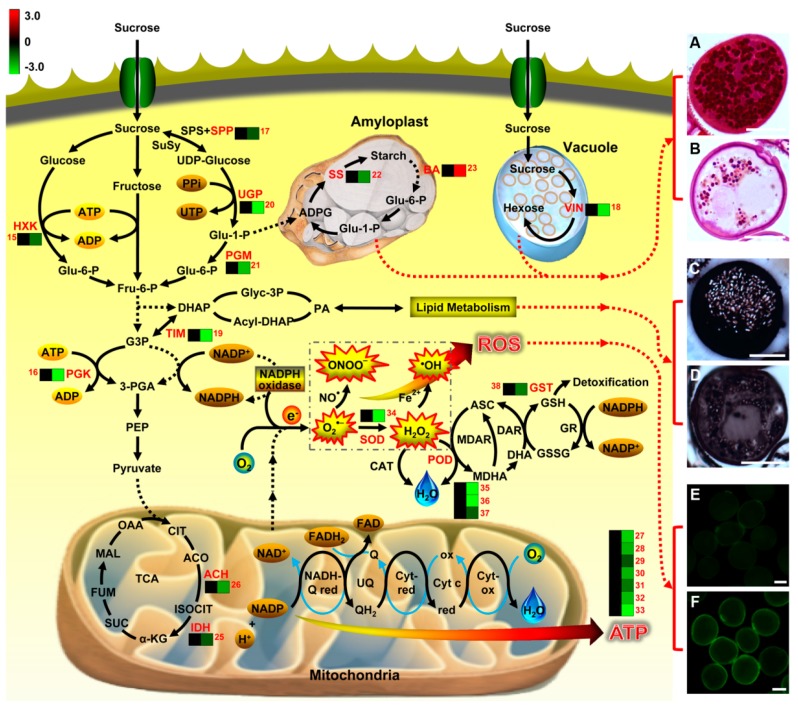
Schematic overview of the metabolic pathways associated with the differentially expressed proteins identified in pollen abortion of CHA-SQ-1-treated wheat plants. Hierarchical clustering and numbers represent protein identification and abundance listed in Tables S1 and S2. SPS, sucrose phosphate synthase; SPP, sucrose phosphate phosphatase; SuSy, sucrose synthase; UGP, UTP-glucose-1-phosphate uridylyltransferase; HXK, hexokinase; Glu-1-P; glucose-1-phosphate; PGM, phosphoglucomutase, Glu-6-P, glucose 6-phosphate; Fru-6-P, fructose 6-phosphate; G3P, glycerate 3-phosphate; DHAP, dihydroxyacetone phosphate; PGK, 3-phosphoglycerate kinase; 3-PGA, 3-phosphoglyceric acid; PEP, phosphoenolpyruvate; TIM, triosephosphat-isomerase; CIT, citrate; ACO, aconitate; ACH, aconitate hydratase; ISOCIT, isocitrate; IDH, isocitrate dehydrogenase; α-KG, α-ketoglutarate; SUC, succinate; FUM, fumarate; MAL, malate; OAA, oxaloacetate; red, reductase; ox, oxidase; UQ, ubiquinone; Q, quinone; QH_2_, hydroquinone; Cyt, cytochrome; ADPG, ADP-glucose; SS, starch synthase; BA, beta-amylase; VIN, vacuolar invertase; Glyc-3P, glyceraldehyde 3-phosphate; PA, phosphatidic acid; SOD, superoxide dismutase; CAT, catalase; POD, peroxidase; MDHA, Monodehydroascorbate; MDHR, Monodehydroascorbate reductase; ASC, ascorbate; DHA, dehydroascorbate; DAR, dehydroascorbate reductase; GSH, reduced glutathione; GSSG, oxidized glutathione; GR, glutathione reductase; GST, glutathione S-transferase. (**A**–**F**) Comparison of insoluble polysaccharides, lipids, and ROS in pollen grains from control (**A**,**C**,**E**) and CHA-SQ-1-treated wheat plants (**B**,**D**,**F**) during pollen maturation. (**A**,**B**) PAS staining. (**C**,**D**) Sudan black B staining. (**E**,**F**) H_2_DCF-DA staining. Bars: 20 μm.

BiNGO indicated statistically over- and under-represented biological pathways related to pollen grains of plants with CHA-SQ-1 treatment (Figure 5; Table S7), which provide a complete list of enriched Gene Ontology (GO) biological pathways of these proteins. Of them, two major biological categories were significantly overrepresented in pollen grains of plants with CHA-SQ-1 treatment, including: metabolic process (*P*= 8.25 × 10^−5^) and response to stimulus (*P*= 8.51 × 10^−6^). This indicates that some of biological pathways in pollen grains responded to resistance and susceptibility conditions under the CHA-SQ-1 treatment, especially oxygen and reactive oxygen species metabolic process (*P*= 9.46 × 10^−4^) and responded to oxidative stress (*P*= 3.96 × 10^−5^), which implied that the pollen grains of CHA-SQ-1 treated plants suffered from oxidative stress (Figure 6E,F). Of them, several of the most highly enriched DAPs were found to participate in carbohydrate metabolism, such as carbohydrate catabolic process (*P*= 2.70 × 10^−5^), hexose metabolic process (*P*= 3.91 × 10^−4^), alcohol metabolic process (*P*= 2.64 × 10^−4^), etc. These results suggested that CHA-SQ-1 disturbed pollen development by several biological pathways, particularly carbohydrate metabolism, oxidative/antioxidative system. More importantly, these abnormal changes in pollen grain of CHA-SQ-1 treatment results in blocking of the process of glucose metabolism and accumulation of starch grains maintaining pollen grain development (Figure 1E,K).

Observations of the connectivity of proteins in this biological network collectively suggest that functional regulation of the cellular mechanisms of pollen abortion in response to CHA-SQ-1 treatment may be involved in significant physiological changes.

## 3. Discussion

### 3.1. CHA-SQ-1 Induced Complete Pollen Abortion

Commercial wheat hybrids have been produced using CHAs, which are growth regulators selectively interfering with the development of pollen or natural systems of male fertility [3,4]. In the present study, the cytological observations indicated that various pollen constituents occurred in CHA-SQ-1-treated plants were markedly different from those in control plants, including abnormal chromosome behavior (Figure S2A,B), low viability (Figure S2C,D), reduced insoluble polysaccharides (Figure 6A,B) and lipid particles (Figure 6C,D), and other storage materials (proteoplasts, starch; Figure 1D–K). These results confirmed that CHA-SQ-1 could induce complete (100%) pollen abortion. Indeed, CHA-SQ-1-induced male sterility is constantly used in China to produce heterosis in wheat [2,4,28]. More importantly, CHA-induced male sterility, with exact the same nuclear background, may circumvent the confounding factors of genotype in cytoplasmic male sterility and genetic male sterility [4], this provides a shortcut for revealing the mechanism of male sterility.

### 3.2. Proteins Involved in Cell Growth and Division

During normal development, the production of functional pollen grain is heavily dependent on timely cell growth and division. In the present study, 11 low-abundant proteins were found to be associated with cell growth and division under CHA-SQ-1 treatment (Table S2).

Mature pollen grains have an outer coat over the underlying wall. The pollen coat composition is critical for protection against environmental damage, such as lipids, phenolic compounds and several other proteins. In addition, some of the components are allergenic to humans, especially pollen allergens. Recent studies showed that pollen allergens serve the plant by fostering vegetative growth and play a role in reproductive development, which were clustered in more than 10 categories [34]. It is important to note that most allergenic pollen proteins are located inside the pollen. Previous studies indicated that CHA-SQ-1 impacts anther development through preceding programmed cell death, misshaping and shrinking extine pattern, and disturbing microspore development [2,31]. In this study, the amount of five pollen allergen proteins (spots 4–8) were significantly decreased in response to CHA-SQ-1 treatment, which resulted in defective pollen intine (Figure 1F,L), leading to membrane and pollen damage, cellular content spilling in pollen grain of CHA-SQ-1 treated plants.

In addition, three DAPs (spots 1–3) were identified as pollen-specific protein in the present study, which are only expressed in a temporally and regionally specific manner and synthesized after microspore mitosis, and then accumulated in the cytoplasm during pollen maturation [35]. Previous studies indicated that pollen-specific *F8-1* is a positive regulator of CHA-SQ-1-induced male sterility, and knockdown of this gene in wheat resulted in 43.56 percent of pollen sterility [34]. Therefore, changes of the structure or pattern of pollen-specific protein can render pollen development abnormal. The data presented here showed that low-abundant of pollen-specific proteins are observed in pollen grains of CHA-SQ-1 treated plants.

### 3.3. Proteins Involved in Carbohydrate and Energy Metabolism

A large proportion (37%) of the proteins was found to be related to carbohydrate and energy metabolism. Of these, 10 proteins were found to be associated with glycolysis (spots 12–21), three with the storage protein (spots 22–24), two with the TCA cycle (spots 25 and 26), and seven with the electron transport chain (spots 27–33; Figure 6 and Table S2).

Mature pollen grains store polysaccharides, lipids, proteins, hormones, and other substances that play important roles in pollen germination and early tube growth. Therefore, it is critical that pollen grains contain sufficient supplies of carbon and energy reserves to utilize at appropriate time [36]. Recent studies indicate that the starch content in CHA-SQ-1 treated anthers was approximately 45% of those in the fertile line; and the activities of vacuolar invertase (VIN) were significantly reduced [28,31]. Meanwhile, the expression of one sucrose transporter gene (TaSUT1) was decreased in CHA-SQ-1 treated anther [28]. Additionally, abscisic acid (ABA)- or cold-induced male sterility in rice involved a disruption of sugar transport in anthers [37]. In this case, sucrose synthesis and degradation play important roles in pollen maturation, germination and tube growth. In both photosynthetic and storage cells, sucrose synthesis involves two enzymes, sucrose phosphate synthase (SPS) and sucrose phosphate phosphatase (SPP). SPP catalyzes the final step in the pathway of sucrose biosynthesis [38], which decreased in abundance and might slow down the process of sucrose biosynthesis of CHA-SQ-1 treated plants (spot 17, Figure 6). Meanwhile, two glycoside hydrolyses (spots 13 and 14) were high-abundant in CHA-SQ-1 treatment, which accelerated the degradation of sucrose. Sucrose is consumed via glycolysis to provide energy necessary for cell expansion, division, differentiation, nutrient uptake, and maintenance during plant development [4]. This requires a series of enzymes to catalyze this process, including Hexokinase (HXK, spot 15), 3-phosphoglycerate kinase (PGK, spot 16), vacuolar invertase (VIN, spots 18), triosephosphat-isomerase (TIM, spots 19), UTP-glucose-1-phosphate uridylyltransferase (UGP, spots 21), and phosphoglucomutase (PGM, spots 21). Of those, as shown in Figure 6, the first step of glycolysis is the phosphorylation of glucose by HXK. PGK, as a major enzyme of glycolysis, catalyzes the first ATP-generating step. TIM is an enzyme that interconverts dihydroxyacetone phosphate and D-glyceraldehyde-3-phosphate very quickly. Its catalytic site located at the dimer interface [39]. UGP1 is an enzyme associated with glycogenesis, and its product, UDP-glucose, is involved in multiple pathways and is a precursor for other sugar nucleotides [36]. PGM is an enzyme that transfers a phosphate group and facilitates the interconversion of glucose 1-phosphate and glucose 6-phosphate. Additionally, glucose and fructose can be produced by VIN in vacuole; in situ hybridization revealed that VIN were highly expressed in the pollen grains [40]. Previous studies have demonstrated that decreased expression of VIN altered the hexose-to-sucrose ratio and VIN has long been believed to be a major player in cell expansion [28]. In the present study, in CHA-SQ-1 treatment, the decreased expression of these proteins (spot 15, spot 16, and spots 18–21) might repress the glycolysis process and further slowed down the pyruvate production rate of glycolysis (Figure 6). Pyruvate is a key intersection of metabolic pathway network, which can be converted to acetyl CoA by the mitochondria pyruvate dehydrogenase complex (mtPDC). The acetyl CoA could enter the TCA cycle and mitochondrial electron transport chain (mtETC), and went through a series of enzyme-catalyzed reaction (such as aconitase, isocitrate dehydrogenase, ATPase, etc.) to supply energy. However, the decreased co-expression of isocitrate dehydrogenase (NAD) regulatory subunit 1 (spot 25), aconitate hydratase (spot 26), and ATP synthase (spots 27–33) further limited the energy production and storage (Figure 6). Therefore, these cause a serious imbalance, and followed by a decrease rate of respiration and ATP production in anthers at trinucleate stage (Figure S3). Taken together, these physiological alterations occur upon disruption of the mitochondrion.

Pollen maturation requires accumulation of starch, which acts as an energy reserve to facilitate pollen germination. For this reason, starch content can serve as a marker of pollen maturity [35]. Recent studies of carbohydrate metabolism indicate that disruption of sugar balance in the pollen grain can impair pollen development significantly and cause male sterility [41]. Here, two key enzymes (spot 22, low-abundant; spot 23, high-abundant) involved in sucrose-to-starch showed different expression patterns in CHA-SQ-1 treated plants (Figure 6), which caused reduced or invisible starch grains (Figure 1K and Figure 6), and resulted in non-functional pollens.

Taken together, the data collected here show that the different expression patterns of 22 proteins inhibited the carbohydrate and energy metabolism, which reduced the level of ATP and sucrose/starch. This indicates that pollens of CHA-SQ-1-treated plants are in a state short of nutrient and energy. With respect to high ATP- and sucrose-requiring processes during pollen maturation, there is not sufficient storage carbohydrates and energy to support cell metabolism. The effects of pollen abortion are likely to be more severe during the period of intense growth.

### 3.4. Stress Response Related Proteins

Proteins included in this group are associated with stress response related proteins, which included eight redox homeostasis- and defense-related proteins (spots 34–41, low-abundant), three signal transduction-related proteins (spot 55, high-abundant; spots 56 and 57, low-abundant). Pollen grains are free-floating and subject to various abiotic stresses, including drought and harsh temperatures. These stresses are often inextricably linked with reactive oxygen species (ROS). To some extent, ROS act as signal molecules in the regulation of biological processes such as growth, development, and responses to both biotic and abiotic stimuli [16,42]. However, excessive ROS production could cause oxidative damage [3,4]. Therefore, pollen grains have evolved a strategy to combat the ROS by inducing various protective enzymes and developing a balance between ROS production and clearance. In our results, many enzymes involved in the ROS detoxification were identified in wheat pollen, including superoxide dismutase (spot 34), peroxidase (spots 35 and 36), L-ascorbate peroxidase (spot 37), and glutathione-S-transferase (spot 38). These antioxidative proteins were low-abundant in CHA-SQ-1-treated pollen grains (Figure 6; Tables S1 and S2), promoting the accumulation of ROS. These conditions led to the occurrence of severe oxidative stress during pollen maturation (Figure 6E,F).

### 3.5. Proteins Related to Synthesis, Folding, and Proteolysis

In the present study, a total of eight DAPs were found to be involved in protein metabolism. Of these, three spots (spots 42–44) corresponding to proteasome involved in protein degradation were identified (Table S2). The other five proteins involved in protein synthesis such as three mitochondrial-processing peptidase subunits (spots 45–47) and two HSPs (spots 48–49) were also identified (Table S2). Other proteomic studies have shown that the same groups of proteins were also identified suffer from abiotic stress during pollen development [43,44]. Therefore, protein synthesis and degradation are important for pollen development. However, in CHA-SQ-1 treated pollen grains, although the process of protein synthesis was enhanced by the increased expression of mitochondrial-processing peptidase subunits (spots 45–47) and HSPs (spots 45–47), it was clearly not sufficient for the accelerated protein degradation caused by the increased expression of proteasome (spots 42–44). Ultimately, CHA-SQ-1-treated pollen grains exhibited an increase protein catabolism (Figure 1J).

### 3.6. Other Proteins

It was documented that DNA methylation reactions happened in CHA-SQ-1-treated anthers [7], and the similar phenomenon was observed in our previous work [32]. In this study, five high-abundant proteins (spots 50–54) associated with one carbon metabolism were identified in CHA-SQ-1-treated pollen grains, which might promote the DNA methylation reactions. Although 2-DE combined with mass spectrometry is likely to provide an extremely useful tool for identifying abundant proteins, there is also some limitation for identifying alkaline and hydrophobic proteins, and some identified proteins might be grouped as unknown proteins due to the limited information on their putative functions [45]. In this study, three spots (spots 58–60) were attributed to proteins with no clearly predicted, well-defined function. This is consistent with the findings of other pollen proteomic studies [14,46].

## 4. Materials and Methods 

### 4.1. Plant Material and Treatment

Wheat plants were treated as described previously [4,32]. CHA-SQ-1 was administered to the wheat cultivar ‘Xinong 1376’ at the rate of 5.0 kg/ha starting when the plants averaged 8.5 on the Feekes’ scale [4]. Control plants received an equal volume of water as a negative control. Pollen grains at trinucleate stage were collected from at least 1500 anthers from 100 plants by tapping the anthers on glass slides. This stage was checked as described previously [2,4]. The samples were examined under a dissecting microscope. Debris was removed with a fine needle. Sampled pollen grains were either used immediately or stored at −80 °C for later use. Three biological replicates were performed. Pollen development was assessed using 1% acetocarmine and fertility with 2% iodine-potassium iodide (2% I_2_-KI) [2]. The activities of respiratory and ATPase were determined according to Wang et al. [4].

### 4.2. Sample Fixation and Infiltration

Pollen grains were fixed immediately in FAA (70% alcohol: 37% formaldehyde: acetic acid; 18:1:1, *v*/*v*/*v*) and performed for light microscopic observation. Pollen grains treated with 2.5% (*v*/*v*) glutaraldehyde were ready for ultrastructural microscopy observation, respectively.

### 4.3. Light Microscopic Observation

Pollen samples were first embedded in Epon-812 resin; semithin (1 μm) sections were cut using an Ultracut E ultramicrotome (Leica Microsystems, Wetzlar, Germany). Then they were stained with Ehrlich’s hematoxylin and safranin O/fast green. The staining method for proteins, lipids, and insoluble polysaccharides was performed according to Konyar and Dane [47] and Tian et al. [48]. The procedure was described briefly as follows.

For Coomassie Brilliant Blue (CBB) staining, semi-thin sections of pollen gains were stained with CBB-R250 solution containing 45% (*v*/*v*) methanol, 10% (*v*/*v*) glacial acetic acid, 45% (*v*/*v*) water and 0.3 g CBB-R250, and then rinsed in distilled water. Proteins were stained blue.

For Sudan black B reaction, semi-thin sections were stained by 70% (*v*/*v*) alcohol saturated with Sudan black B, then rinsed in a mixture of 70% (*v*/*v*) alcohol and distilled water. This caused the lipids in solution to turn black.

A periodic acid-Schiff (PAS) reaction was performed, in which transverse sections were oxidized in 1% (*w*/*v*) periodic acid and stained for 1 h in Schiff’s reagent. They were then destained in 0.5% (*w*/*v*) sodium bisulfite. This caused the insoluble polysaccharides to turn pink and purple.

Samples were imaged with a DS-U2 high-resolution camera and Nikon ECLIPSE E600 microscope equipped with the NIS-Elements software (Nikon, Tokyo, Japan).

### 4.4. Electron Microscopic Observation

For transmission electron microscopy (TEM), ultrathin sections (50 to 70 nm) were produced using a UC6 ultramicrotome (Leica, Wetzlar, Germany) and collected on copper grids. They were then double-stained in 2% (*w*/*v*) aqueous uranyl acetate and 2.6% (*w*/*v*) aqueous lead citrate. Finally, they were examined under a HT7700 transmission electron microscope (Hitachi, Tokyo, Japan).

### 4.5. Fluorescence Microscopic Observation

Then 4′,6-diamidino-2-phenylindole (DAPI; Sigma-Aldrich, Oakville, Ontario, Canad) was used to stain nuclei, and a fluorescein diacetate (FDA; Sigma-Aldrich, Oakville, Ontario, Canad) assay was performed to assess the vitality of fresh pollen grains. Samples were washed, embedded, and stained as described previously [2].

For ROS detection, fresh pollen grains were washed with PBS, then incubated in 5 µM H_2_DCF-DA (Sigma-Aldrich) dissolved in anhydrous dimethyl sulfoxide (DMSO; Sigma-Aldrich) for 60 min in the dark at 25 °C. Excess probe was removed using PBS before detection. 

Fluorescent signals were captured using a fluorescence microscope (Olympus BX 51, Olympus, Japan). Filter sets for blue (DAPI, 4′,6-diamidino-2-phenylindole) and green fluorescence (FDA, H_2_DCF-DA) were Olympus part numbers.

### 4.6. Protein Extraction

The pollen protein was extracted with TCA/acetone methods described by Song et al. [30] and Zhang et al. [49] with modifications. Briefly, pollen gains were ground to fine powder in liquid nitrogen. This powder was then suspended in −20 °C pre-cooled solution of 10% TCA, 0.07% β-ME and 1 mM PMFS and kept at −20 °C overnight. The samples were centrifuged at 20,000× *g* for 20 min at 4 °C, and the pellets were rinsed with a pre-cooled acetone solution containing 0.07% (*v*/*v*) β-ME and 1 mM PMSF. They were then centrifuged at 25,000× *g* for 30 min. Three rounds of rinsing and centrifugation were performed. Then the vacuum-dried pellets were dissolved in lysis solution containing 2 M thiourea, 7 M urea, 65 mM DTT, 4% (*w*/*v*) CHAPS, and 0.5% (*v*/*v*) Bio-Lyte (Bio-Rad, Hercules, CA, USA) and 0.001% (*w*/*v*) bromophenol blue. Insoluble materials were centrifuged out, and protein concentration was determined using a Bio-Rad Protein Assay Kit II (Bio-Rad, Hercules, CA, USA) according to the manufacturer’s instructions.

### 4.7. Two-Dimensional Gel Electrophoresis

2-DE was performed as described by Song et al. [30] and Wang et al. [4]. Approximately 550 μg of protein was separated by loading the sample onto a 17 cm pH 4–7 linear pH gradient IPG strip (Bio-Rad, Hercules, CA, USA). The sample was then subjected to electrophoresis on the IPGphor apparatus (Protean IEF Cell; Bio-Rad, Hercules, CA, USA) at 80 kV-h, and the conditions were as follows: constant power (50 μA/IPG strip) at 250 V for 1 h, 500 V for 1h, 1000 V for 1 h, 8000 V for 4 h and 8000 V for a total of 80,000 V-h (17 cm, pH 4–7). The second electrophoretic dimension was performed using 12% SDS-PAGE. The gels were stained with CBB-G250. There were three independent biological replicates per sample.

### 4.8. Image Analysis

The stained gels were imaged using a PowerLook 2100XL scanner (UMAX, Taiwan, China) and analyzed using PDQuest 2-DE 8.0.1 (Bio-Rad, Hercules, CA, USA). Briefly, the images were initially processed through transformation, filtering, automated spot detection, normalization, and matching. All protein spots detected in gel were matched to the corresponding spots of master gel (the control), and then each spot density was normalized against the whole gel densities. Analysis was based on total densities of gels as percent volume, and spots were considered to have significant differences in expression if the mean abundance changed more than 1.5-fold (*P* < 0.05) as indicated by the *t* test.

### 4.9. MS Analysis and Database Search

DAPs detected in stained gels were selected manually and excised for protein identification. In-gel digestion of DAPs was described by Song et al. [30] and Wang et al. [4]. Specifically, it was performed after destaining, reduction, and alkylation, and executed by incubation at 37 °C for 16 h in trypsin solution. All samples were subjected to MALDI-TOF/TOF mass spectroscopy on a 5800 MALDI Time of Flight (TOF)/TOF^TM^ analyzer (AB Sciex, Foster City, CA, USA). Mass spectra were acquired by TOF/TOF^TM^ Series Explorer^TM^ Software (version 4.1, AB Sciex) that recorded across a range of mass from 700 to 4000 Da with a focus on 1700 Da. For one main MS spectrum 15 sub-spectra with 200 shots per sub-spectrum were accumulated, and for the MS/MS spectrum up to 25 sub-spectra with 250 shots per sub-spectrum were accumulated.

All of the MS/MS data were checked against the NCBInr database using the Mascot search engine (www.matrixscience.com) with the taxonomy parameter set to green plants. Other parameters were as follows: enzyme of trypsin, one missed cleavage site, fixed modification of carbamidomethyl (Cys), variable modification of Gln->pyro-Glu (N-term Q) and oxidation (Met), peptide tolerance of 100 ppm, MS/MS tolerance of 0.3 Da, and peptide charge of 1+.

For positive identification, the peptides were considered to be identified when the scoring value exceeded the identity or extensive homology threshold score of identity value, as calculated by Mascot, based on the MOWSE score. Sequences of proteins identified as unknown, hypothetical, or of uncharacterized function served as queries in a search for their homologues using the BLASTP algorithm. The mass spectrometry proteomics data have been deposited to the ProteomeXchange Consortium via the PRIDE [50] partner repository with the dataset identifier PXD012519.

### 4.10. Bioinformatic Analysis of Identified Proteins

The details of bioinformatics analysis have been described previously [30,32]. The DAPs defined by MS analysis were classified using the gene index and Uniprot accession number, which were entered into the Uniprot database (http://www.uniprot.org). Hierarchical clustering was performed on log 2-transformed data using Genesis 1.7.6 software. All identified proteins were blasted against *Arabidopsis thaliana* TAIR10 protein databases (http://www.arabidopsis.org/). The protein–protein interaction network was analyzed using STRING 10, which is publicly available (http://string-db.org/). Biological processes were predicted using the Cytoscape plugin BiNGO 3.02. Subcellular localization of the identified proteins was determined using Euk-mPLoc 2.0 database (http://www.csbio.sjtu.edu.cn/bioinf/euk-multi-2/), BaCelLo database (http://gpcr.biocomp.unibo.it/bacello/pred.htm) and ESLpred database (http://www.imtech.res.in/raghava/eslpred2/).

## 5. Conclusions

In seed plants, pollen grains transport sperm cells to the female gametophyte. In this study, cytological and proteomic changes of wheat pollen abortion induced by CHA-SQ-1 were investigated. The cytological study indicated that pollen grain was impaired by CHA-SQ-1 during its maturation, which appeared as abnormal pollen development and shape, reduced storage materials, and defective pollen wall. A total of 60 identified DAPs with various functions were identified in mature pollen grains; some of these are central actors in biological processes (carbohydrate metabolism and energy metabolism) that regulate pollen maturation, especially some proteins related to sucrose and starch metabolism and ROS metabolism (Figure 5 and Figure 6). Ultimately, these results induce complete (100%) pollen abortion in CHA-SQ-1-treated plants. Hence, this study has investigated the cytological, physiological and biochemical changes during pollen maturation in CHA-SQ-1-treated plants, which could provide a valuable resource for plant biology research, particularly for sexual reproduction in plants.

## Supplementary Materials

Supplementary materials can be found at https://www.mdpi.com/1422-0067/20/7/1615/s1. Figure S1. The replicate 2-DE electrophoresis gels of pollen grain proteomes in the control (A, C) and CHA-SQ-1-treated wheat plants (B, D). Figure S2. Comparison of nuclei and vitality in pollen grains from control (A, C) and CHA-SQ-1-treated wheat plants (B, D) during pollen maturation. (A, B) DAPI staining. (C, D) FDA staining. Bars: 20 μm. Figure S3. Analysis of respiratory activity and ATPase activity. (A) Total respiration (V_t_), and activities of the cytochrome pathway (V_cyt_) and the alternative pathway activity (V_alt_) in control and CHA-SQ-1-treated wheat plants. (B) Analysis of ATPase activity. Data are means ± SD of three independent experiments (biological replicates). The significant of differences was assessed by Student’s *t*-test (**P* < 0.05, ***P* < 0.01). Table S1. Folds change of differential abundance proteins (DAPs) in pollen grain between the control and CHA-SQ-1-treated wheat plants. Table S2. Identification of differentially expressed proteins. Table S3. Matched peptide sequences of identified proteins. Table S4. Corresponding homologues of the three unknown proteins. Table S5. Differentially expressed proteins blasted against the TAIR database. Table S6. Abbreviations of the specific protein names in the STRING network (Figure 6). Table S7. Biological pathways of differentially expressed proteins.

## Abbreviations

2-DE	Two-dimensional gel electrophoresis
CBB	Coomassie Brilliant Blue
CHA	Chemical hybridizing agent
DAPI	4′, 6-diamidino-2-phenylindole
DAPs	Differential abundant proteins
DMSO	Dimethyl sulfoxide
FDA	Fluorescein diacetate
HXK	Hexokinase
mtETC	Mitochondrial electron transport chain
mtPDC	Mitochondria pyruvate dehydrogenase complex
PAS	Periodic acid-Schiff
PGK	3-phosphoglycerate kinase
PGM	Phosphoglucomutase
pI	Isoelectric point
ROS	Reactive oxygen species
SPP	Sucrose phosphate phosphatase
SPS	Sucrose phosphate synthase
TEM	Transmission electron microscopy
TIM	Triosephosphat-isomerase
UGP	UTP-glucose-1-phosphate uridylyltransferase
VIN	Vacuolar invertase
