# Supplementary figures and images for: Cytological and Proteomic Analysis of Wheat Pollen Abortion Induced by Chemical Hybridization Agent
Source: Int J Mol Sci. 2019 Apr 1;20(7):1615. doi: 10.3390/ijms20071615 (PMC6480110; doi:10.3390/ijms20071615)

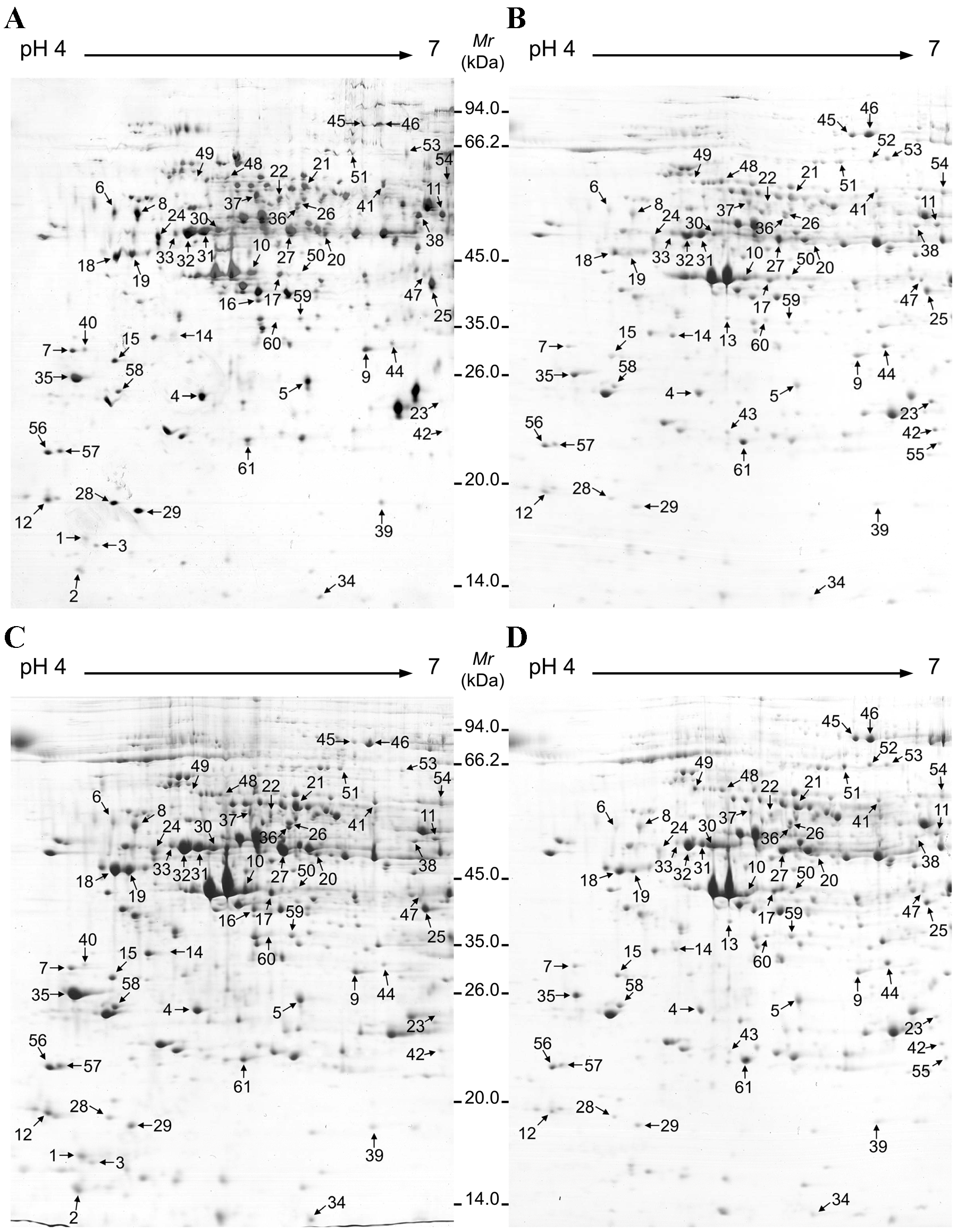

Supplement: Supplementary file 1 [file ijms-20-01615-s001.zip › Supplementary Files/Figure S1.tif]

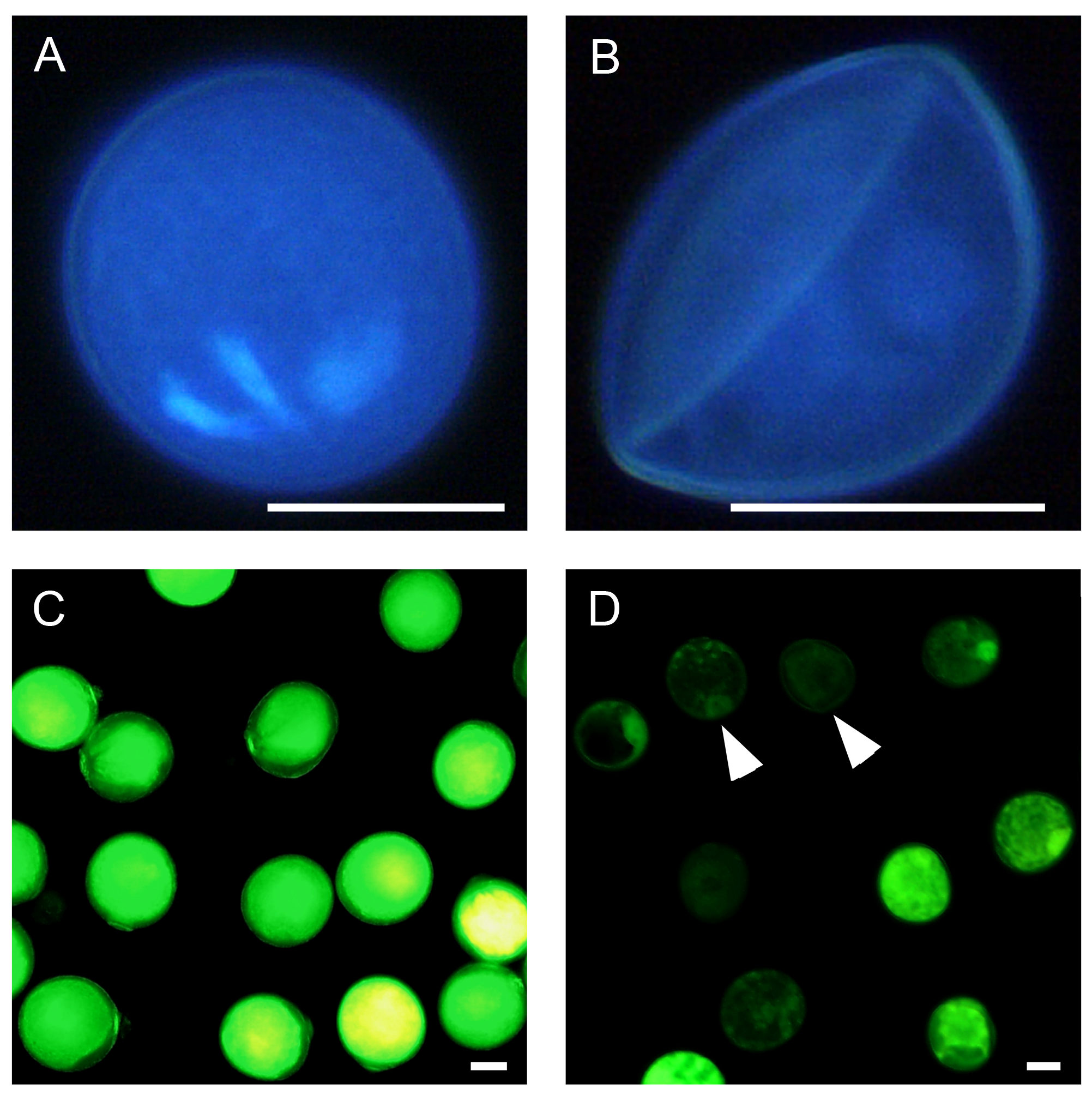

Supplement: Supplementary file 1 [file ijms-20-01615-s001.zip › Supplementary Files/Figure S2.tif]

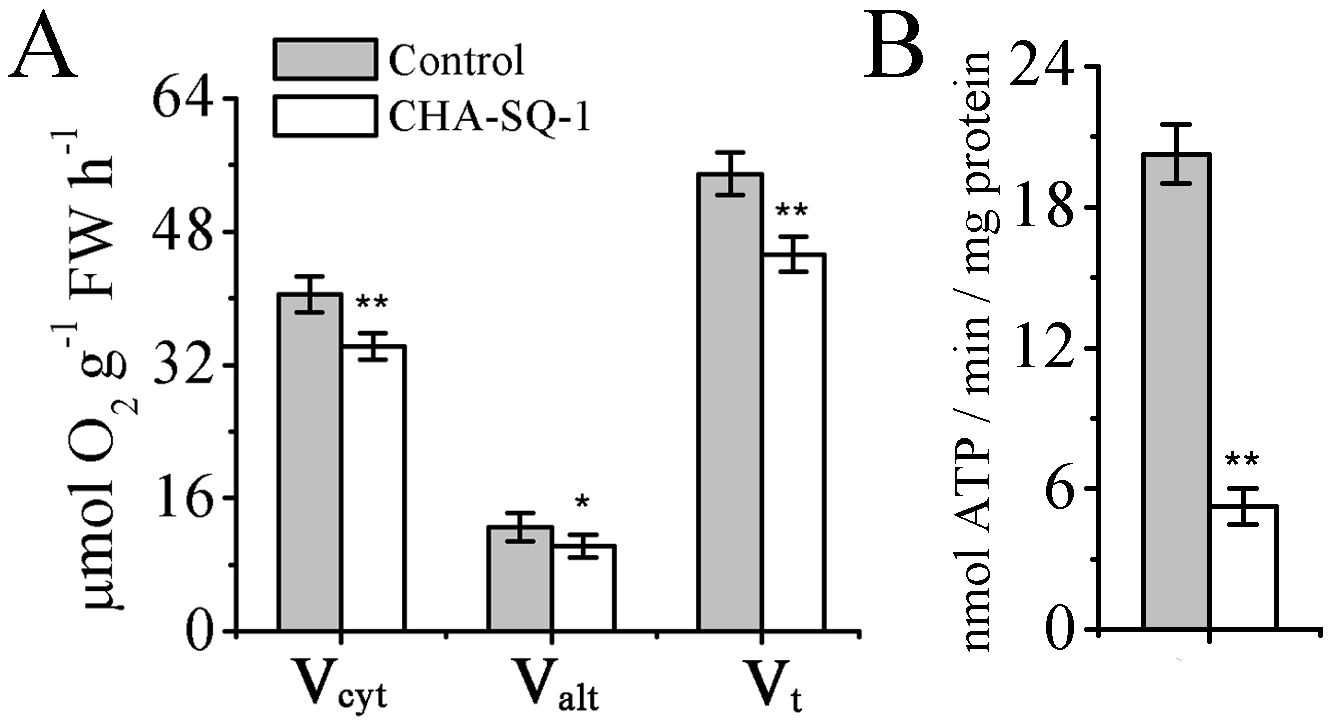

Supplement: Supplementary file 1 [file ijms-20-01615-s001.zip › Supplementary Files/Figure S3.tif]
